# Omental torsion

**DOI:** 10.4103/0971-9261.44770

**Published:** 2008

**Authors:** Paresh Jain, Sheri Chhabra, Ketan Parikh, Amrish Vaidya

**Affiliations:** Department of General Surgery, Jaslok Hospital and Research Centre, Mumbai, India

**Keywords:** Acute abdomen, appendicitis, omental torsion

## Abstract

Omental torsion is a rare cause of acute abdominal pain, and clinically mimics acute appendicitis. A 11-year-old boy presented with symptoms and signs suggestive of appendicitis. A computed tomography of abdomen revealed findings suggestive of omental torsion. Diagnostic laparoscopy confirmed the diagnosis of torsion of a segment of the greater omentum.

## INTRODUCTION

Torsion of the omentum is a condition wherein the organ twists along its long axis to such an extent that its vascularity is compromised. Although omental torsion is rarely diagnosed preoperatively, knowledge of the entity is important to the surgeon because it mimics the common causes of acute surgical abdomen.

## CASE REPORT

**Figure 1:**
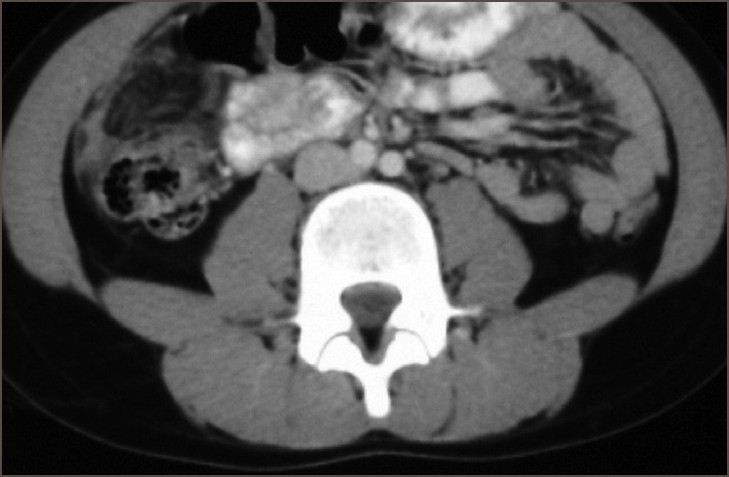
CT scan showing fat stranding anterior to Cecum and ascending colon in RIF

An 11-year-old boy presented with right-sided abdominal pain of four days duration. There was no history of any surgical interventions in the past. Physical examination revealed tenderness in right lumbar region, with mild guarding. The general examination was normal. Hernial orifices and the genitalia were also normal. Ultrasound examination revealed an echogenic mass in the right lumbar region, anterior to the colon, with the appearance of abnormal fatty tissue. The right iliac fossa appeared normal. Blood investigations showed a total leukocyte count of 4780/cmm with predominant neutrophilia, and the other laboratory tests were within normal limits. A CT scan revealed fat stranding anterior to the ascending colon in right lumbar region with normal findings in the region of the appendix [Figure 1]. Laparoscopy revealed gross inflammation of parietal peritoneum over anterior abdominal wall. A segment of greater omentum, measuring 12 × 10 × 2 cm, was rotated around its longitudinal axis at the base. The omentum distal to the twisted segment was discolored, edematous, inflamed, and adherent to the anterior abdominal wall. Minimal free fluid in the pelvis was noted, but the appendix could not be visualized. A segmental omentectomy was done. The postoperative period was uneventful. The histopathology report was suggestive of acute omentitis with areas of hemorrhage and necrosis.

## DISCUSSION

Omental torsion is a rare pathology. Patients commonly present with right iliac fossa pain resembling the pain associated with appendicitis, but often lacking the associated gastrointestinal symptoms of nausea, vomiting, or anorexia. Kimber et al, reviewed over 8000 cases of appendectomies, quoting omental torsion as being the finding in one out of every 600 operations for presumed appendicitis, when the appendix was found to be normal.[1]

Omental torsion can be primary or secondary. Primary torsion is unipolar with one end of the omentum free while secondary torsion is bipolar where the end opposite to the vascular pedicle is fixed to adhesions or some other pathological condition. Primary torsion is said to occur when there is no pathological cause found, and normally occurs in fourth or fifth decade of life. It has also been described in children above the age of four years, probably, due to the increase in omental fat deposition as the child grows.[2] The cause of primary omental torsion remains unknown. Redundancy of the omental veins as compared to the arteries, results in venous engorgement of Rupture of a dependent vein, precipitating thrombosis has been proposed as an etiological factor. Spitz *et al*, suggested multiple predisposing factors like changes in omental consistency including inflammation, edema, and excess fat deposition (obesity) or anatomic malformations including tongue-like projections and bifid and accessory omentum.[3] Secondary omental torsion is always associated with abdominal pathology including tumors and cysts, postsurgical scarring, and hernia sacs. In one of the study, it was shown that it often torts around the right epiploic artery.[2]

Clinically, primary and secondary omental torsions are similar. The most frequent complaint is pain in the right iliac fossa, which is sudden in onset and, at times, may be associated with nausea, vomiting, and low-grade fever. A past history of a similar but less severe pain may be present. Goti et al, stated that 66% of these cases mimic appendicitis, and 22%, cholecystitis.[4]

Ultrasonography may show a complex mass and mixture of solid material and hypoechoic zones and free fluid within the peritoneal cavity. On the other hand, CT scan is very sensitive for showing an omental mass but not specific for making a diagnosis of torsion. Classical signs of omental torsion on CT scan are of a hazy fatty mass with concentric linear strands in the greater omentum, the whirl sign. These strands are twisted blood vessels whirling around a central rod. However, there are other differential diagnoses of hazy fatty mass with associated stranding, such as omental hernia, inflammation of epiploic appendages, paniculitis, and fat-containing neoplasms.

As far as treatment is concerned, it can be conservative and is expectant in stable patients.[5] This includes reassurance, analgesics, and antibiotics, and resolution is expected in two weeks. However, though initially conservatively managed, our patient had persisting tenderness and fever and hence, surgery was planned. With the advent of laparoscopy, omental torsion is being visualized easily, and the chances of missing the pathology at surgery are now rare. In our case, diagnosis was made by CT scan and laparoscopy confirmed the pathology. Treatment involves resection of the diseased segment of omentum and to correct any secondary pathology, if present. It has been observed that, if the omentum is not excised it may become atrophic and fibrotic and, on rare occasions, the pedicle may even autoamputate, leading to automatic clinical regression.[6] Also, at times in case of untreated situation, one may see omental necrosis because of hemorrhagic infarction, intra-abdominal abscesses, peritonitis, or bowel obstruction.[7,8] Spontaneous derotation may be possible and may explain omental adhesions found during laparotomy that have no clear cause. Hence, laparoscopy is the best method of diagnosis, therapy and to shorten the course of the disease.

Primary omental torsion is an entity that mimics many acute abdominal pathologies and hence should be included in the differential diagnosis. In cases, where, imaging may not help, laparoscopy can be used as a diagnostic and therapeutic tool.
